# Ganglioside-Dependent Neural Stem Cell Proliferation in Alzheimer’s Disease Model Mice

**DOI:** 10.1177/1759091415618916

**Published:** 2015-12-22

**Authors:** Noah A. Koon, Yutaka Itokazu, Robert K. Yu

**Affiliations:** 1 Department of Neuroscience and Regenerative Medicine, Medical College of Georgia, Augusta University, Augusta, GA 30912, USA; 2 Charlie Norwood VA Medical Center, Augusta, GA 30904, USA

**Keywords:** Alzheimer’s disease, amyloid β-peptide, glycosyltransferase, glycogene, neural stem cell, cell proliferation

## Abstract

The aggregation and formation of amyloid plaques by amyloid β-peptides (Aβs) is believed to be one of the pathological hallmarks of Alzheimer’s disease (AD). Intriguingly, Aβs have also been shown to possess proliferative effects on neural stem cells (NSCs). Many essential cellular processes in NSCs, such as fate determination and proliferation, are heavily influenced by cell surface glycoconjugates, including gangliosides. It has recently been shown that Aβ1-42 alters several key glycosyltransferases and glycosidases. To further define the effects of Aβs and to clarify the potential mechanisms of action of those peptides on NSCs, NSCs were cultured from embryonic brains of the double-transgenic mouse model of AD [B6C3-Tg(APPswe,PSEN1dE9)85Dbo/J] coexpressing mutants of amyloid precursor protein (APPswe) and presenilin1 (PSEN1dE9). We found that Aβs not only promoted cell proliferation but also altered expression of several key glycogenes for glycoconjugate metabolism, such as sialyltransferases II and III (ST-II & -III) in AD NSCs. In addition, we found upregulation of epidermal growth factor receptor and Notch1 intracellular domain. Moreover, the increased expression of ST-II and -III coincided with the elevated levels of c-series gangliosides (A2B5+ antigens) in AD NSCs. Further, we revealed that epidermal growth factor signaling and gangliosides are necessary components on Aβ-stimulated NSC proliferation. Our present study has thus provided a novel mechanism for the upregulation of c-series ganglioside expression and increases in several NSC markers to account for the proliferative effect of Aβs on NSCs in AD mouse brain. These observations support the potential beneficial effects of Aβs and gangliosides in promoting neurogenesis in AD brain.

## Introduction

Alzheimer’s disease (AD) is the most prevalent form of dementia and a severe neurodegenerative disorder with clinical symptoms that include deficits in memory, judgment, thinking, and behavior. These symptoms usually develop slowly and become worse over time. They interfere with daily tasks and ultimately lead to death. It is well accepted that deposition of aggregated amyloid β-peptide (Aβ) to form amyloid plaques, also known as senile plaques, together with associated reactive astrocytosis and dystrophic neuritis, represent major pathological hallmarks of AD (Selkoe, 1997, 2013). The most studied physiologically relevant Aβs include 4-kDa peptides, including two dominant forms Aβ1-40 and Aβ1-42. Aβs are derived from proteolytic cleavage of amyloid precursor protein (APP). The two dominant forms of Aβs have a high tendency to assemble initially into the soluble form and later to insoluble aggregated fibrils as extracellular amyloid plaques in the AD brain. Intermediate soluble oligomers of Aβs, rather than the aggregated Aβs, are increasingly recognized as having cellular toxicity in AD (Kuo et al., 1996; Lansbury, 1999; Hardy and Selkoe, 2002; Lesne et al., 2006; Liu et al., 2015). Genetic linkage analyses of familial cases of AD have identified APP and presenilins as the highest risk factors for AD pathogenesis (Guerreiro et al., 2013). During normal metabolism of amyloid protein (APP), Aβ1-40 is abundantly produced. However, mutations of APP and presenilins elevate the secretion of Aβs, especially the more abundant form Aβ1-42 (Selkoe, 1999, 2013). Approximately 1% of freshly dissolved monomeric Aβ1-40s forms dimers, but under the same conditions, about 7.4% of Aβ1-42 is converted to the dimeric form (Roher et al., 1996). This observation suggests that the soluble form of Aβ1-42 has a higher tendency to exist as a dimer. Aβ dimers are the most abundant form of soluble oligomers in the cortex of AD patients (Jin et al., 2011). Aβs are produced by cultured cells as part of the normal cellular metabolism (Haass et al., 1992; Seubert et al., 1992). Because Aβ1-40 and Aβ1-42 are present in the brain and cerebrospinal fluid of normal individuals, it suggests that these peptides may possess certain physiological activities in normal life (Shoji, 2002). Despite extensive efforts for studying Aβs for their cytotoxic effects in AD, the normal biological functions and positive effects of Aβs have remained elusive. With respect to their impact on neural stem cells (NSCs), we and others have reported that Aβ1-42 promoted NSC proliferation (Lopez-Toledano and Shelanski, 2004; Heo et al., 2007; Sotthibundhu et al., 2009; Diaz-Moreno et al., 2013; Itokazu et al., 2013; Itokazu and Yu, 2014), presumably due to the fact that Aβ1-42 stimulated Notch signaling and upregulated the expression of fucosyltransferase-IX (FUT9), which is a key enzyme for the synthesis of the Lewis x carbohydrate epitope (Itokazu and Yu, 2014).

Gangliosides are sialic acid-containing glycosphingolipids expressed primarily in the outer leaflet of the plasma membrane of all vertebrate cells and are particularly abundant in the nervous system (Ngamukote et al., 2007; Yu and Itokazu, 2014; Yu et al., 2012). During cellular differentiation and brain development, it is known that dramatic and consistent changes in the composition of neural gangliosides occur (Yu et al., 1988; Ngamukote et al., 2007). In rodent brain, a shift from the synthesis of simple gangliosides, such as GM3 and GD3, to the synthesis of the more complex gangliosides in the a- and b-series, particularly GM1, GD1a, GD1b, and GT1b, during brain development has been well documented. A2B5 monoclonal antibody was reported in 1979 (Eisenbarth et al., 1979), and it recognizes c-series gangliosides, including GQ1c, GT1c, and GT3 (Kasai and Yu, 1983; Saito et al., 2001). The c-series gangliosides are abundant in embryonic mammalian brains but are present in extremely low amount in adult brains (Ngamukote et al., 2007). Stage-specific embryonic antigen-1 (SSEA-1/Lewis X/CD15) is a well-known carbohydrate antigenic epitope of undifferentiated cells and has been recognized as an NSC marker (Yu and Itokazu, 2014). The 3-fucosyl-*N*-acetyllactosamine or Lewis X carbohydrate structure is defined as [Galβ1-4(Fucα1-3)GlcNAcβ-]. Lewis X carbohydrate epitope is synthesized by transferring a fucosyl residue from GDP-fucose to *N*-acetylglucosamine (GlcNAc) by the action of α1,3-FUT9 (Kudo et al., 1998). Recently, we showed that FUT9 knockdown in mouse NSCs impaired Musashi-1 expression and NSC proliferation, suggesting that NSC proliferation can be modulated by FUT9 and the Notch signaling pathway (Yagi et al., 2012).

At present, there has not been any effective therapy to halt the progression of AD, although it is generally accepted that it is more promising to halt the progression of AD during the earlier stages (Bateman, 2015). With the advent of stem cell therapy, it is expected that the use of NSCs may contribute to the treatment of AD as well as several other neurodegenerative disorders. NSCs are undifferentiated neural cells characterized by the capacity for self-renewal and proliferation with retention of multipotency, that is, generating brain-forming cells such as neurons, astrocytes, and oligodendrocytes. For the therapeutic use of NSCs in AD, a detailed clarification of the effects of Aβs and gangliosides on NSCs is warranted. In this study, we investigated the possible interactions between gangliosides and NSC proliferation in AD brain. Here, we demonstrated that NSCs from embryonic brains of a double-transgenic mouse model of AD have increased capacity for proliferation with upregulation of key glycogenes for c-series gangliosides synthesis, such as sialyltransferases II and III (ST-II and-III). Both are key enzymes involved in the synthesis of c-series gangliosides whose expression is characteristic of NSCs. The increased expression of ST-II and -III elevated levels of c-series gangliosides (A2B5-antigen). In AD NSCs, NSC-associated markers, such as epidermal growth factor receptor (EGFR) and Notch1 intracellular domain (NICD), were upregulated. Interestingly, our results revealed that EGF and b/c-series gangliosides are required for Aβ-stimulated NSC proliferation. Our present study thus provides a novel mechanism for NSC proliferation in AD brain tightly regulated by gangliosides and suggests that Aβs and gangliosides could be used as potential therapeutic targets for promoting neurogenesis in AD brain.

## Materials and Methods

### Materials

Aβ1-42 was purchased from Bachem Americas (Torrance, CA). Freshly prepared soluble Aβs were used for the experiment. Antibodies used were as follows: anti-Notch-1 (mouse; BD Biosciences, San Jose, CA), anti-EGFR (rabbit; Santa Cruz Biotechnology, Dallas, TX), and antiactin (rabbit; Sigma-Aldrich, St. Louis, MO).

### NSC Culture

AD mice [B6C3-Tg(APPswe,PSEN1dE9)85Dbo/J] (Jankowsky et al., 2001) were purchased from the Jackson Laboratory (Bar Harbor, ME), and ST-II-knockout (KO) mice were originally provided by the courtesy of Dr. Richard Proia (NIDDK, NIH, Bethesda, MD). Wild-type (WT) littermates of these mice were used for control. Mouse NSCs were prepared from embryonic brains in the form of neurospheres, which were floating clonal aggregates formed by NSCs *in vitro* (Nakatani et al., 2010). In brief, single-cell suspensions were prepared from the striata of embryonic day (E)-14.5 mouse brains. NSCs were cultured in Neurobasal A medium (Life Technologies, Carlsbad, CA) supplemented with B27 (Life Technologies) and 20 ng/ml of fibroblast growth factor 2 (FGF2; Peprotech, Rocky Hill, NJ) and 20 ng/ml of EGF (Peprotech). Neurospheres formed after 1 week were collected for further passages and analyses. The use of animals for this study was approved by the Institutional Animal Care and Use Committees at Georgia Regents University and the VA Medical Center, Augusta, GA.

### WST-8 Assay

The number of cultured NSCs was estimated by the WST-8 assay using a Cell Counting Kit-8 (Dojindo, Kumamoto, Japan). The dissociated NSCs from neurospheres were plated at a density of 1 × 10^4^ cells per well onto 96-well plates that had been coated with poly-l-ornithine (Sigma-Aldrich) and fibronectin (Sigma-Aldrich). After 3 days of culture, 10 μl of WST-8 solution was added to each well. After incubating for 3 hr in a CO_2_ incubator, the spectrophotometric absorbance of WST-8-formazan produced by the dehydrogenase activity in the living neural cells was measured at the wavelength of 450 nm using a Benchmark Plus Microplate Spectrophotometer (Bio-Rad Laboratories, Hercules, CA). The spectrophotometric absorbance measured by this assay was highly correlated with the number of living NSCs (Kanemura et al., 2002; Itokazu et al., 2013; Itokazu and Yu, 2014). For adding Aβ1-42, Aβs freshly dissolved in media were replaced to each well on the next day after cell plating, and WST-8 analysis was performed on those cells after 3 days.

### Reverse Transcription-Polymerase Chain Reaction

Total RNA samples were isolated from cultured NSCs using the Trizol reagent (Life Technologies). cDNAs were synthesized based on the total RNAs as templates using MultiScribe™ Reverse Transcriptase (Applied Bioscience). PCR was performed using ReadyMix™ REDTaq® (Sigma-Aldrich) with the following settings: 94℃ for 5 min; 26 to 40 cycles of 94℃ for 30 s, 60℃ for 30 s, and 72℃ for 30 s; and 72℃ for 5 min. PCR products were analyzed by agarose gel electrophoresis using 1.5% agarose gels containing SYBR Safe™ DNA Gel stain (Life Technologies). The bands were quantified using the NIH ImageJ 1.46 r image processing program (rsb.info.nih.gov) to reflect the original mRNA levels. Densitometric data were normalized against β-actin mRNA. The normalized value from control (WT) is defined as 1.0. Primer sequences are described elsewhere (Ngamukote et al., 2007; Nakatani et al., 2010; Itokazu and Yu, 2014).

### Western Blotting

NSCs were washed with phosphate-buffered saline (PBS), lysed in radioimmunoprecipitation assay buffer containing 50 mM Tris-HCl, 150 mM NaCl, 5 mM NaF, 1 mM Na_3_VO_4_, 1% NP-40, 0.5% sodium deoxycholate, and 1% sodium dodecyl sulfate, pH 7.5, supplemented with a complete protease inhibitor cocktail (Roche Applied Science, Indianapolis, IN), and then centrifuged at 12,000 × *g* and 4℃ for 10 min. Supernatants (cell lysates) were collected, and the protein concentrations were measured using a bicinchoninic acid protein assay kit (Thermo Fisher Scientific Inc., Rockford, IL). Proteins were separated by sodium dodecyl sulfate polyacrylamide gel electrophoresis (8% gel) under reducing conditions and transferred to polyvinylidene difluoride membranes. The membranes were probed with primary antibodies followed by appropriate secondary antibodies conjugated with horseradish peroxidase (BD Biosciences). Signals were visualized with Western Lightning Western blot chemiluminescence reagent (Perkin Elmer Life and Analytical Sciences, Boston, MA). The bands were quantified using the NIH ImageJ. Densitometric values were normalized by setting the NICD/actin or EGFR/actin protein ratio for control (WT) in each treatment. The normalized value from control (WT) is defined as 1.0.

### Immunocytochemistry

Cells were fixed in 4% paraformaldehyde for 20 min at room temperature, washed three times with PBS, and blocked with 5% goat serum and 1% BSA for 30 min. The treated cells were then incubated with antibodies overnight in a chamber with a humid atmosphere at 4℃. The primary antibody used was an anti-A2B5 mouse antibody (IgM). After incubation with the primary antibody, the cells were washed three times with PBS and incubated with Alexa 488 anti-mouse IgM antibody (Thermo Fisher Scientific Inc.) for 2 hr in the dark, washed and then incubated with 4,6-diamidino-2-phenylindole (Thermo Fisher Scientific Inc.) for 5 min to stain the nuclei. Specimens were mounted in Fluoro-Gel from Electron Microscopy Sciences (Hatfield, PA) and observed using a Zeiss LSM 510 confocal microscope (Carl Zeiss GmbH, Jena, Germany).

### Statistical Evaluation

Data are expressed as means ± standard error of the mean from 3 to 20 independent experiments. Statistical significance was determined using one-way analysis of variance followed by Tukey’s post hoc multiple comparison test and unpaired two-tailed Student’s *t* test, and *p* < .05 was regarded as significant.

## Results

### Increased Number of NSCs in AD Mouse Brain

We have reported that supplemented Aβ1-42 promotes mouse NSC proliferation (Itokazu et al., 2013; Itokazu and Yu, 2014). In the previous study, to evaluate cell death accompanied by DNA fragmentation, terminal deoxynucleotidyl transferase-mediated dUTP nick-end labeling (TUNEL) assay was performed after Aβ treatments. It has revealed that there were no significant TUNEL-positive cells in the intact cells and NSCs treated with Aβs (Itokazu et al., 2013). That result was also in accord with our previous report showing that the expression of active caspase 3 was not different in intact cells and NSCs treated with the soluble form of Aβs (Yanagisawa et al., 2010). Those results suggest that the increase of NSC number upon treatment with Aβs was likely caused by cell proliferation rather than by protection the cells from undergoing apoptosis.

**Figure 1.**
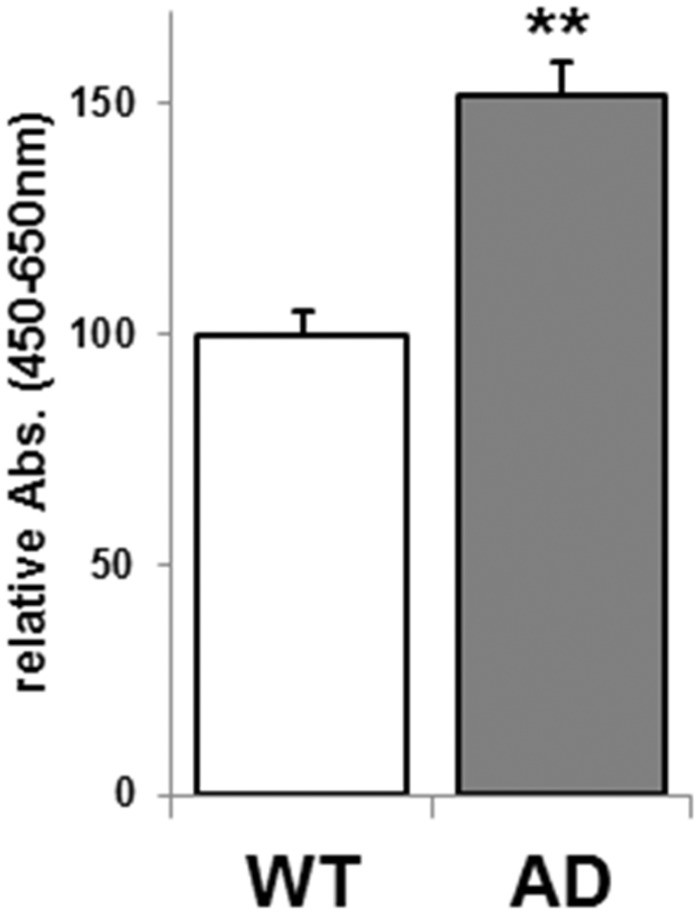
Increased number of NSCs in AD mouse *in vitro*. The number of NSCs from wild-type (WT) and AD mice [B6C3-Tg(APPswe,Psen1dE9)85Dbo/J], cultured for 3 days as monolayer culture with FGF2 and EGF, was estimated by the WST-8 assay. The *y* axis represents the relative absorbance (Abs.), which indicates the percentage of absorbance against WT. Each bar represents mean ± *SEM* of 20 independent experiments (*n* = 20). Comparison was made between AD mice versus their WT littermates. **p < .01. AD = Alzheimer’s disease; NSCs = neural stem cells; FGF = fibroblast growth factor; EGF = epidermal growth factor.

NSCs were cultured from embryonic brains (E 14.5) of the double-transgenic mouse model of AD coexpressing mutants of APP (APPswe) and presenilin1 (PSEN1dE9). The dissociated NSCs (from neurospheres) were plated at a density of 1 × 10^4^ cells/well onto 96-well plates, coated with poly-l-ornithine and fibronectin. After 3 days of culture, the number of NSCs was estimated by the WST-8 assay. The absorbance measured by this assay was highly correlative with the number of living NSCs (Kanemura et al., 2002). NSCs from AD mouse model (AD NSCs) significantly increased the number of cells compared with their littermate WT (Figure 1). Recently, it was reported that all of the NSCs from this AD mice are immunopositive for anti-Aβ monoclonal antibody (6E10) and that Aβ monomers (Aβ1-40 and 1-42) and a wide range of oligomers are detected in both cell lysate and culture media of AD NSCs, but these peptides are not expressed in NSCs of their WT littermates (Ghate et al., 2014).

### Expression of NSC-Associated Markers

**Figure 2.**
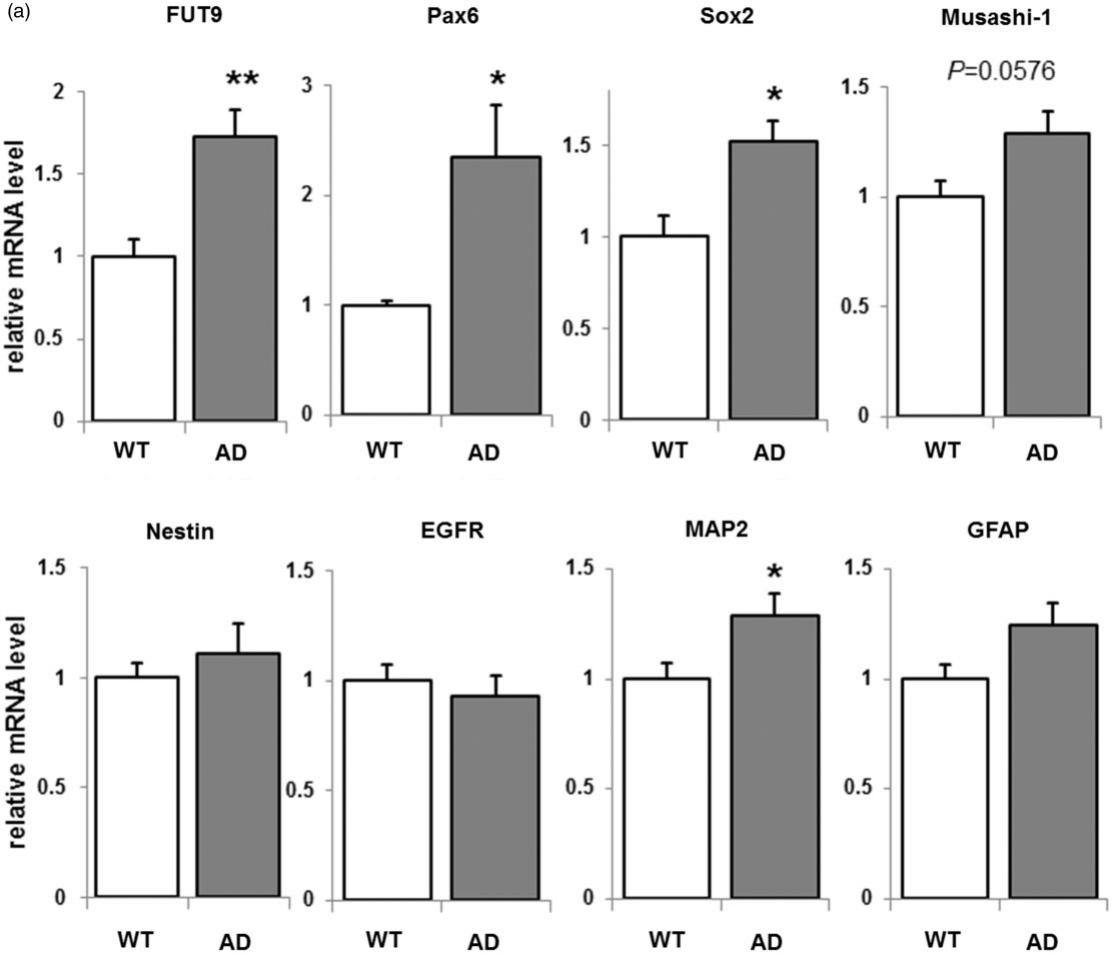

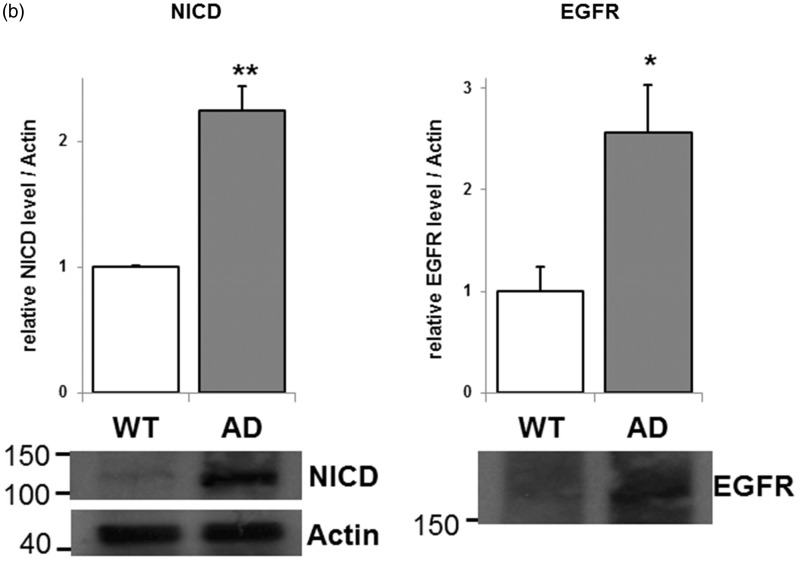
Expression of neural cell lineage-associated markers in AD NSCs. (a) NSCs from AD mice were cultured for 3 days. RT-PCR analyses were performed using specific primer sets. β-actin was used as a control. RT-PCR products were resolved on agarose gels, and the intensity was quantified by normalization against β-actin mRNA. The normalized value from WT is defined as 1.0. Each bar represents mean ± *SEM* of three to five independent experiments (*n* = 3–5). *p < .05 and **p < .01 indicate the level of significance in two-tailed *t* tests of differences between AD mice versus their WT littermates. (b) Western blot of NSCs from AD mice and immunostained using anti-Notch1, anti-EGFR, and antiactin antibodies. Values were normalized by setting the Notch1 intracellular domain (NICD)/actin or EGFR/actin protein ratio for WT as 1. Each bar represents mean ± *SEM* of three independent experiments (*n* = 3). *p < .05 and **p < .01 indicate the level of significance in two-tailed *t* tests of differences between AD mice versus their WT littermates. WT = wild-type; AD = Alzheimer’s disease; NSCs = neural stem cells; RT-PCR = reverse transcription-polymerase chain reaction; EGFR = epidermal growth factor receptor; MAP2 = microtubule-associated protein 2; GFAP = glial fibrillary acidic protein; FUT9 = fucosyltransferase-IX.

Many essential cellular processes, such as fate determination and proliferation, in NSCs are profoundly influenced by cell surface glycoconjugates whose expression is dramatically altered during differentiation. FUT9 is a key enzyme for the synthesis of Lewis X-carrying N-glycans, which has been used as an NSC marker (Yu and Itokazu, 2014). In our previous study, FUT9 was shown to increase following treatment by Aβ1-42, which is expected to affect NSC proliferation (Itokazu and Yu, 2014). For this reason, we analyzed the gene expression level of FUT9 as well as cell lineage-associated markers in AD NSCs (Figure 2(a)). The expression of FUT9 increased in AD NSCs. FUT9 was reported to be controlled by Pax6 that promotes proliferation of neural progenitor cells and neurogenesis, and it was revealed that Pax6 mRNA expression was increased in AD NSCs. Other NSC markers, such as Musashi-1 and SOX2, were also upregulated in AD NSCs. On the other hand, the expression of the mRNA of a glial marker (glial fibrillary acidic protein [GFAP]) was not significantly altered. These are consistent with the fact that Aβ1-42 stimulated FUT9, Pax6, and Musashi-1 expression as shown by our reverse transcription-polymerase chain reaction (RT-PCR) experiment in a previous study (Itokazu and Yu, 2014). The expression of the mRNA of a neuronal marker (microtubule-associated protein 2) is also elevated.

Because we previously showed that NICD was increased in Aβ1-42-stimulated proliferating NSCs (Itokazu and Yu, 2014), we performed Western blot analyses to examine the protein expression of Notch (Figure 2(b)). Western blot analysis shows that the protein expression of NICD was upregulated in AD NSCs. In addition to the Notch signaling pathway, the EGFR pathway is also important to regulate stem cell proliferation. It is known that both pathways are capable of regulating the NSC number and self-renewal capability (Aguirre et al., 2010). We also examined the protein expression level of EGFR and found that the EGFR expression was significantly elevated in AD NSCs (Figure 2(b)). These data are consistent with the notion that both Notch and EGFR pathways are contributing to the enhanced cell proliferation for AD NSCs.

### Key Glycosyltransferase and c-Series Ganglioside Expressions

**Figure 3.**
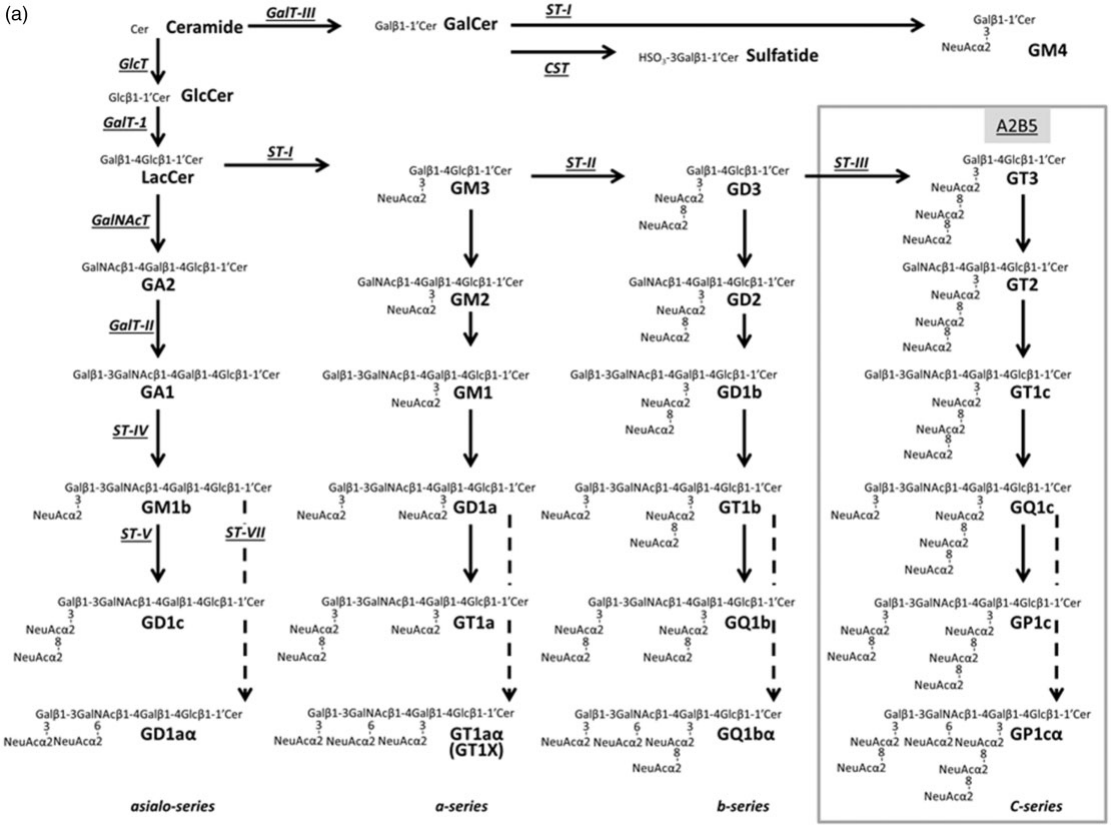

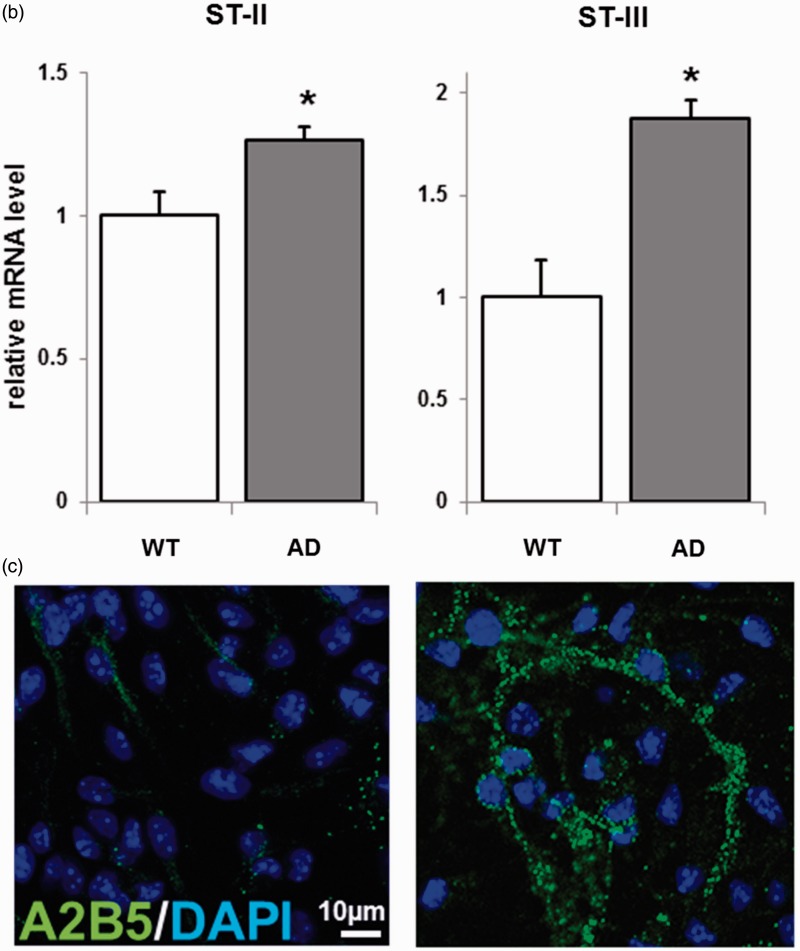
Metabolic pathways and key glycosyltransferases for glycosphingolipids, including gangliosides. (a) The nomenclature for gangliosides and their components are based on those of Svennerholm (1963) and IUPAC-IUBMB Joint Commission on Biochemical Nomenclature (1976). β-gal = lysosomal acid β-galactosidase; GalNAc-T = N-acetylgalactosaminyltransferase I (GA2/GM2/GD2/GT2-synthase); GalT-I = galactosyltransferase I (lactosylceramide synthase); GalT-II = galactosyltransferase II (GA1/GM1/GD1b/GT1c-synthase); GLCC = glucosylceramidase; GlcT = glucosyl transferase (glucosylceramide synthase); GM2A = GM2 activator protein; HEX, β-N-acetylhexosaminidase; ST-I = sialyltransferase I (GM3-synthase); ST-II = sialyltransferase II (GD3-synthase); ST-III = sialyltransferase III (GT3-synthase); ST-IV = sialyltransferase IV (GM1b/GD1a/GT1b/GQ1c-synthase); ST-V = sialyltransferase V (GD1c/GT1a/GQ1b/GP1c-synthase); ST-VII = sialyltransferase VII (GD1aα/GT1aα/GQ1bα/GP1cα-synthase). (b) NSCs from AD mice were cultured for 3 days. RT-PCR analyses were performed for sialyltransferases II and III (ST-II and -III), two key glycosyltransferases for synthesis of c-series gangliosides whose expression is characteristic of NSCs. RT-PCR products were resolved on agarose gels, and their intensities were expressed as ratios by normalization against β-actin mRNA. The normalized value from WT is defined as 1.0. Each bar represents mean ± *SEM* of four independent experiments (*n* = 4). *p < .05 indicates the level of significance in two-tailed *t* tests of differences between AD mice versus their WT littermates. (c) c-Series gangliosides (A2B5+, green fluorescence) were significantly increased in AD NSCs. Images were processed identically for the two micrographs shown for each fluorophore so that intensities can be compared. WT = wild-type; AD = Alzheimer’s disease; DAPI = 4,6-diamidino-2-phenylindole; NSCs = neural stem cells; RT-PCR = reverse transcription-polymerase chain reaction.

ST-II and -III are key glycosyltransferases for synthesis of c-series gangliosides whose expression is characteristic of NSCs (Figure 3(a)). We performed RT-PCR experiments and semiquantified gene expressions of ST-II and -III. We found increased expression of ST-II and -III in AD NSCs (Figure 3(b)). Next, we evaluated the expression of A2B5 antigens, including c-series gangliosides, by immunocytochemistry. The increased expression of ST-II and -III resulted in elevated levels of c-series gangliosides (A2B5-antigens) in AD NSCs (Figure 3(c)). These data suggest that c-series gangliosides may also be associated with NSC proliferation in AD.

### EGF Is Required for Increased NSC Proliferation in AD

**Figure 4.**
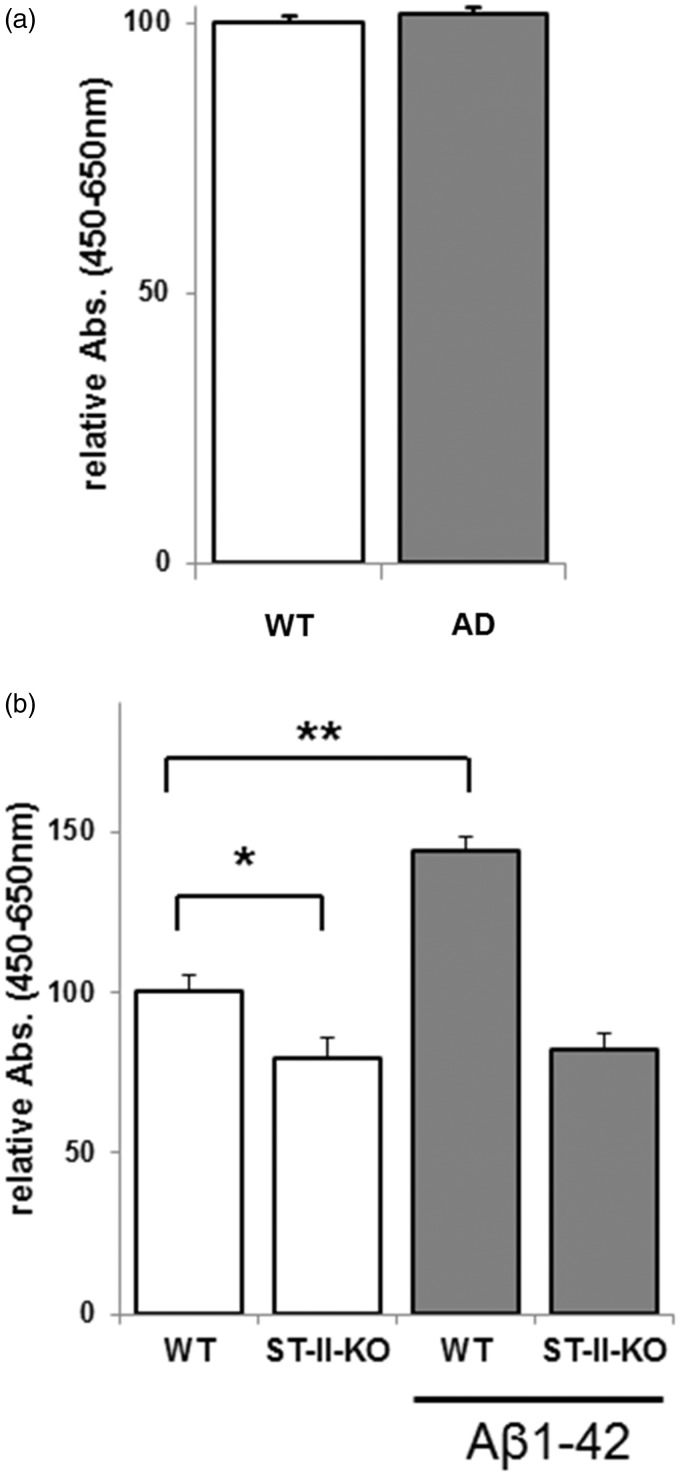
EGF signaling is required for cell proliferation of AD NSCs, and gangliosides are necessary for Aβ-stimulated NSC proliferation. (a) NSCs were isolated from brains of AD mice and their WT littermates. NSCs were cultured for 3 days with FGF2 but without EGF, and the number of NSCs was estimated by the WST-8 assay. The *y* axis represents the relative absorbance (Abs.), which indicates the percentage of absorbance against WT. Each bar represents mean ± *SD* of 12 independent experiments (*n* = 12). Comparison was made between AD mice versus their WT littermates. No statistical significance was detected on the number of NSCs in this condition (−EGF, +FGF2). (b) NSCs were isolated from ST-II (GD3 synthase)-KO mice and their WT littermates. NSCs were cultured for 3 days with or without Aβs. The number of NSCs in the monolayer culture in the presence of soluble Aβ1-42 (10 μM) was estimated by the WST-8 assay. The *y* axis represents relative absorbance (Abs.), which represents the percentage of absorbance against vehicle treatment. Each bar represents mean ± *SD* of eight independent experiments (*n* = 8). *p < .05 and **p < .01 indicate the level of significance. WT = wild-type; AD = Alzheimer’s disease; ST-II = sialyltransferase II; KO = knockout; EGF = epidermal growth factor; NSCs = neural stem cells; FGF = fibroblast growth factor.

We found that the expression of EGFR was significantly increased in AD NSCs (Figure 2(b)). We previously reported that ST-II-KO NSCs that were cultured with EGF showed significantly suppressed cellular proliferation (Wang and Yu, 2013). Surprisingly, however, no difference in the proliferation rate and expression of lineage-associated markers was found between ST-II+/+ (WT) NSCs and ST-II-KO NSCs that were cultured in the presence of FGF2 but in the absence of EGF (Yu and Yanagisawa, 2007). Accordingly, this ganglioside-dependent NSC proliferation is considered to be regulated mainly through the EGFR signaling pathway. To investigate whether the increase of cell proliferation in AD NSCs was dependent EGFR-signaling, we cultured AD NSCs in the absence of EGF. It was revealed that AD NSCs did not show any increased cell proliferation under this condition (Figure 4(a)). Because FGF2 is known to maintain NSC proliferation even in the absence of EGF (Yanagisawa et al., 2010), the results clearly showed that EGFR signaling is a key pathway for Aβ-induced NSC proliferation.

### Gangliosides Are Required for Aβ-Stimulated NSC Proliferation

The earlier results suggest that the enhanced cell proliferation in AD NSC could depend on gangliosides through the EGFR-signaling (Wang and Yu, 2013). To evaluate this possibility, NSCs were isolated from ST-II-KO mice in which GD3 and its downstream products, including b- and c-series gangliosides, are missing (Figure 3(a)), and their WT littermates. Figure 4(b) shows that Aβ1-42 dramatically increased WT NSC proliferation as similar as shown in Figure 1. Most intriguingly, however, Aβ1-42 could not stimulate cell proliferation on ST-II-KO NSCs. The results clearly show that b- or c-series gangliosides are required for Aβ1-42-induced NSC proliferation.

## Discussion

The present study provides the first direct evidence that gangliosides are required for Aβ-stimulated NSC proliferation. We and others have previously reported that supplemented soluble Aβ1-42-stimulated NSC proliferation. In this study, we discovered that NSCs from an mouse AD model [B6.Cg-Tg (APPswe,PSEN1dE9)85Dbo/J] showed increased cell proliferation with upregulation of ganglioside expression. Recently, NSCs have been cultured from this AD model mouse (Ghate et al., 2014), and these AD NSCs are confirmed to highly express APP and its proteolytic peptides including both Aβ monomer and a wide range of pathogenic oligomers (2 to 14-mers) *in vitro* culture. Aβ peptides (1-40 and 1-42) were detected only in AD NSCs, but they were not expressed in NSCs from their WT littermates. These authors did not find any signs of cytotoxicity in these AD NSCs (Ghate et al., 2014). In our previous study, soluble Aβ1-40 and Aβ1-42 did not affect the number of apoptotic cells on NSCs (Itokazu et al., 2013). Taken together, it suggests that the increase of the number of NSCs in AD was likely caused by enhanced cell proliferation rather than by protection the cells from apoptosis.

Notch and EGFR pathways are important to control stem cell fate determination, and it is known to regulate NSC number and self-renewal via these pathways (Aguirre et al., 2010). We showed that NICD was increased in AD NSCs (Figure 2(b)). Presenilins are a necessary substrate transporter and the catalytic component of γ-secretase (van Tijn et al., 2011). γ-Secretase is a transmembrane enzyme that cleaves APP and catalyzes the formation of Aβ peptides. γ-Secretase also cleaves Notch and releases NICD that is translocated to the nucleus to regulate expression of genes important for development (Hardy and Israel, 1999). The mutation of PSEN1dE9 in AD models lacks the endoproteolytic cleavage site which is located at exon 9 of presenilin1, and this mutant PSEN1dE9 is constitutively active (Thinakaran et al., 1996). It has been reported that Notch expression is upregulated in brains of AD patients (Berezovska et al., 1998; Fischer et al., 2005). Further, Davis et al. (1998) reported that both human WT and AD mutant presenilin1 elevate Notch1 expression and restore normal development in presenilin1-KO mouse. It is therefore possible that the presenilin1 mutation expressed in the Tg(APPswe,PSEN1dE9) mouse model of AD may be responsible for the enhanced Notch cleavages and increased NICD level for NSC proliferation.

EGFR signaling plays central roles in cell proliferation differentiation and neuronal development, and it is known that NSCs express EGFR (Reynolds et al., 1992; Reynolds and Weiss, 1992; Craig et al., 1996; Kornblum et al., 2000; Ramalho-Santos et al., 2002). It has been reported that mice lacking either EGFR or its brain enriched ligand heparin binding EGF-like growth factor (Hb-EGF) die at birth, and the survival strains die by postnatal day 20 to 25 (P20–P25) with severe neurogdegeneration (Miettinen et al., 1995; Sibilia and Wagner, 1995; Threadgill et al., 1995; Iwamoto et al., 2003). Recently, the expression of EGFR was reported to be positively regulated by presenilin1, specifically in neural cells, and presenilin1-knockdown NSCs had reduced EGFR expression, resulting in decreased NSC proliferation (Gadadhar et al., 2011; Bruban et al., 2015). Therefore, it is possible that the presenilin1 constitutive active mutant (PSEN1dE9) enhances EGFR expression on AD NSCs, as shown in the current study (Figure 2(b)). In postmortem AD-patient brain, however, it has been reported that intense EGFR expression was detected at the periphery of plaque formations in the cerebral cortex and hippocampus (Birecree et al., 1988). Thus, it can be considered that EGFR modulates NSC functions in AD brain under physiological and pathophysiological conditions.

Although adult neurogenesis is gradually diminished with the onset of aging in mice, a significant number of NSCs still exists in aged brains (Jin et al., 2003). Not surprisingly, the EGFR expression in rodent brain is also reduced with aging (Hiramatsu et al., 1988). Jin et al. reported that bromodeoxyuridine (BrdU)-labeled cells were reduced by 90% in dentate gyrus (DG) and 50% in SVZ of 20-month-old mice compared with 3-month-old mice. Infusions of Hb-EGF restored and increased the number of BrdU + cells by 450% in DG and 250% in SVZ of 20-month-old mice (Jin et al., 2003). It is considered that Hb-EGF can activate EGFR and induce NSC proliferation via the EGFR signaling pathway (Higashiyama et al., 1991; Kornblum et al., 1999). Recently, Hb-EGF is also reported to be required for maintaining synaptic plasticity and memory function in adult mice (Oyagi et al., 2011). These studies reinforce the notion that EGFR signaling pathway is crucial in maintaining adult NSCs and in retarding the aging process of the brain.

Recently, we presented evidence that GD3, which is the major ganglioside in NSCs (Nakatani et al., 2010), was required for maintaining the self-renewal ability of mouse NSCs *in vivo* and *in vitro* (Wang and Yu, 2013; Wang et al., 2014). In the absence of GD3, NSCs from postnatal ST-II-KO mice had even lower self-renewal capacity than NSCs from embryonic ST-II-KO mice. Our previous *in vivo* study also showed that the ventricular wall was significantly thinner in adult ST-II-KO mouse brain, and there was a progressive loss of NSCs in DG (80%) and SVZ (70%) regions of 6-month-old ST-II-KO mouse brains. In addition, the greater impaired neurogenesis in the adult ST-II-KO mice led to depression-like behaviors in adult animals (Wang et al., 2014). For maintenance of healthy NSC functions, our previous study suggested that GD3 has important roles to keep cell surface EGFR expression by modulating the EGFR intracellular recycling pathway (Wang and Yu, 2013). The expression of EGFR mRNA was not changed (Figure 2(a)), whereas the protein level of EGFR was highly elevated (Figure 2(b)) in AD NSCs. Taken together, gangliosides may enhance the sorting of EGFR through the early endosomes to the recycling endosomal pathway, rather than through the degradative lysosomal pathway in AD NSCs. The present study demonstrates that b-/c-series gangliosides and EGF-signaling are both necessary for Aβ-stimulated NSC proliferation (Figure 4(b)). Accordingly, gangliosides, which are important modulator(s) of EGFR signaling pathway, may maintain adult NSC functions. Further studies are needed to examine whether the EGFR recycling pathway is similarly involved in enhancing NSC proliferation in AD brain.

It has been reported that endogenous neurogenesis is altered in AD patients and transgenic AD mouse models. Recently, stage-specific changes in neurogenesis were studied in AD patients’ brains (Ekonomou et al., 2015). In that study, it was found that there was a trend showing an increase in the number of neuroblasts (PCNA+/HuC/D+) in the DG of moderate AD brains, but eventually a decrease in the number of immature neurons (HuC/D+) in the DG of patients at the stage of severe AD (Ekonomou et al., 2015). The majority of AD cases (>95%) are sporadic, and only 5% of AD cases are explained by genetic mutations (Alzheimer’s Association, 2014). Increased NSC proliferation is also reported in sporadic AD mouse model (Diaz-Moreno et al., 2013).

Regarding the aging process and AD, it is worth mentioning that a senescence-accelerated mouse prone (SAMP8) model of accelerated senescence, which was originally generated from AKR/J mice after selective breeding (Takeda et al., 1981), has been proposed as a suitable model to study changes associated with aging and AD brains, given that SAMP8 mice develop AD lesions in an age-dependent manner (Butterfield and Poon, 2005; Morley et al., 2012). More recently, Díaz-Moreno et al. reported that NSC cultures from 2-month-old SAMP8 mice displayed increased cell proliferation. On the other hand, NSC proliferation was decreased at 10 months of age in the SAMP8 mouse. In their *in vivo* study, the number of BrdU+ proliferating cells was similar in 1-month-old mice, transiently elevated between 2 months (BrdU+ cells increased by almost 2-fold compared with 1 month) and 6 months (about 1.4-fold increased from 1 month) of ages in SAMP8 mice, and finally decreased in older (14-month-old) SAMP8 mice compared with control animals. GFAP+/SOX2+ NSCs were increased significantly at SVZ, and the thickness of the ventricular wall was also significantly larger in 2-month-old SAMP8 mice than that in the control strain (Diaz-Moreno et al., 2013).

In conclusion, the studies from AD patients and mouse models clearly indicate an enhanced neurogenesis in early/moderate stage of AD and then neurogenesis becomes impaired in late/severe AD brains. In late/severe AD brains, neurogenic impairments may underlie, at least in part, the progressive loss of memory and compromised ability to learn and process new information characterizing the disease. Both olfactory and hippocampal dysfunction might be enhanced by compromised neurogenesis in the SVZ and in the DG, respectively. Therefore, endogenous neurogenesis has been suggested as an important treatment target in AD (Mu and Gage, 2011). In our previous study, soluble form of Aβ1-42 or aggregated forms of Aβ1-40 or Aβ1-42 significantly increased the number of adult NSCs as well as embryonic NSCs (Itokazu et al., 2013; Itokazu and Yu, 2014). Although other determinants (e.g., APP intracellular domain, soluble APP beta, or presenilin) that are germane to Aβs may also contribute to enhancement of cell proliferation, our present study provides a novel mechanism for NSC proliferation in AD brain that is tightly regulated by gangliosides. Ganglioside expression profiles are associated not only with central nervous system development but also with the pathogenic mechanisms of central nervous system diseases, such as AD. Understanding the roles of gangliosides and Aβs on NSC functions should be very useful in providing novel strategies for promoting adult neurogenesis in AD damaged brains.
